# Genome‐wide association study of soluble solids content, flesh color, and fruit shape in citron watermelon

**DOI:** 10.1002/tpg2.20391

**Published:** 2023-09-18

**Authors:** Dennis N. Katuuramu, Amnon Levi, William P. Wechter

**Affiliations:** ^1^ USDA‐ARS, U.S. Vegetable Laboratory Charleston South Carolina USA

## Abstract

Fruit quality traits are crucial determinants of consumers’ willingness to purchase watermelon produce, making them major goals for breeding programs. There is limited information on the genetic underpinnings of fruit quality traits in watermelon. A total of 125 citron watermelon (*Citrullus amarus*) accessions were genotyped using single nucleotide polymorphisms (SNPs) molecular markers generated via whole‐genome resequencing. A total of 2,126,759 genome‐wide SNP markers were used to uncover marker‐trait associations using single and multi‐locus GWAS models. High broad‐sense heritability for fruit quality traits was detected. Correlation analysis among traits revealed positive relationships, with the exception of fruit diameter and fruit shape index (ratio of fruit length to fruit diameter), which was negative. A total of 37 significant SNP markers associated with soluble solids content, flesh color, fruit length, fruit diameter, and fruit shape index traits were uncovered. These peak SNPs accounted for 2.1%–23.4% of the phenotypic variation explained showing the quantitative inheritance nature of the evaluated traits. Candidate genes relevant to fruit quality traits were uncovered on chromosomes Ca01, Ca03, Ca06, and Ca07. These significant molecular markers and candidate genes will be useful in marker‐assisted breeding of fruit quality traits in watermelon.

## Core Ideas

- A panel of citron watermelon accessions was extensively phenotyped for fruit quality traits over two field growing seasons.
- Using 125 accessions and 2,126,759 genome‐wide SNP markers, three GWAS models were fitted to discover marker‐trait associations.
- Genomic loci and candidate genes were identified and will be useful in the improvement of watermelon for fruit quality traits.

**Abbreviations**
BLINK: Bayesian‐information and linkage‐disequilibrium iteratively nested keyway
BLUE: best linear unbiased estimate
FarmCPU: fixed and random model circulating probability unification
GWAS: genome‐wide association study
MLM: mixed linear model
PCA: principal component analysis
PVE: phenotypic variation explained
QQ: quantile–quantile
REML: restricted maximum likelihood
SNP: single nucleotide polymorphism
USVL: U.S. Vegetable Laboratory

## 1 INTRODUCTION

Cultivated watermelon (*Citrullus lanatus*) is a popular vegetable crop that is grown and consumed worldwide for its fleshy, sweet, and nutritious fruit (Maoto et al., 2019; Zamuz et al., 2021). Globally, over 101.6 million tonnes of sweet watermelon were produced in 2021 (FAOSTAT, 2022). *Citrullus lanatus* is genetically related to citron watermelon (*Citrullus amarus*) and these two species can be intermated in controlled crosses to introgress alleles controlling traits of interest (Jarret et al., 2017). Centuries of domestication, breeding, and selection efforts focused on yield and fruit quality traits have resulted in narrowing of the genetic base in present‐day sweet‐fleshed watermelon germplasm collections (Levi et al., 2001). *Citrullus amarus* exhibits phenotypic plasticity for a range of traits that are crucial in the improvement of cultivated watermelon (Ali et al., 2012; Katuuramu et al., 2022, 2020; Levi et al., 2013). Priority fruit quality traits for watermelon cultivar development that drive repeat purchase and consumption include fruit size, soluble solids content (sweetness), flesh color, fruit shape, and rind patterns (Maynard, 2001; Wehner, 2008).

Soluble solids content and flesh color are two of the major internal fruit quality characteristics that influence consumer choice (Kaiser, 2012; Maynard, 2001). Fruit flesh color is a prominent quality trait in watermelon with red, yellow, and orange being the most consumed types worldwide (Wehner, 2008). Both soluble solids content and flesh color are polygenically inherited traits (Bang et al., 2010; Hashizume et al., 2003). Broad‐sense heritability for soluble solids content ranging from moderate to high in inter‐specific bi‐parental populations generated from *C. lanatus* and *C. amarus* has been reported (Ren et al., 2014). Genomic regions controlling soluble solids content and flesh color on chromosomes 2, 4, 5, and 10 in *C*. *lanatus* have been reported (Fall et al., 2019; Guo et al., 2019; Wu et al., 2019). Fruit shape is an important horticultural trait in commercial watermelon breeding. Fruit shape can be used to categorize watermelon into market classes as shape greatly influences the number of fruits that can be packed into standard boxes before shipment and marketing of the produce (Kaiser, 2012; Wehner, 2008). The oval or blocky‐oval fruit shape is preferred for packing in standard shipment boxes, especially in the seedless watermelon industry (Maynard, 2001; Wehner, 2008). For diploid watermelon markets and where consumers prefer large fruit size, elongate fruit shape is more common (Kaiser, 2012; Wehner, 2008). Fruit shape is a function of length, diameter, and ratio of length to diameter or fruit shape index. In watermelon, fruit shape can be classified as round, oval, or elongate (UPOV, 2013; Wehner, 2008; Wehner et al., 2001). Fruit shape is quantitatively inherited with genomic regions on chromosomes 1, 2, 3, and 6 reported in previous genetic studies (Dou et al., 2018; Guo et al., 2019; Wu et al., 2019).

The continuous improvement of DNA sequencing platforms and cost reductions have facilitated the development of chromosome‐scale genomes and high‐density molecular markers in watermelon (Guo et al., 2019; Katuuramu et al., 2022; Wu et al., 2019). Availability of these genomic resources has enabled implementation of genome‐wide association mapping to uncover genomic regions and causal polymorphisms underlying economically important traits in watermelon. The genome‐wide association study (GWAS) trait mapping methodology utilizes densely genotyped diverse germplasm with historical meiotic events and high allelic diversity to uncover marker‐trait associations (Ingvarsson & Street, 2011; Korte & Farlow, 2013; Zhu et al., 2008). In this project, 125 citron watermelon accessions were extensively phenotyped for fruit quality traits (soluble solids content, flesh color, and fruit shape) and GWAS was applied to these phenotypes to uncover marker‐trait associations using both single‐ and multi‐locus models.

**Core Ideas**

- A panel of citron watermelon accessions was extensively phenotyped for fruit quality traits over two field growing seasons.
- Using 125 accessions and 2,126,759 genome‐wide SNP markers, three GWAS models were fitted to discover marker‐trait associations.
- Genomic loci and candidate genes were identified and will be useful in the improvement of watermelon for fruit quality traits.

## 2 MATERIALS AND METHODS

### 2.1 Plant materials, cultural practices, and experimental design

A total of 125 citron watermelon accessions were received from the USDA germplasm repository in Griffin, GA, and used in this research. This germplasm has been described and used previously in a downy mildew‐citron watermelon patho‐system genetic study (Katuuramu et al., 2022). The genotypes were grown in the field over 2 years (2019 and 2022) at the U. S. Vegetable Laboratory (USVL) research site located in Charleston, SC. The soil type is mostly Yonges loamy fine sand with a pH of 6.0, organic matter of 0.9%, and cation exchange capacity of 4.7 meq 100 g^−1^. In both years, the field site was double‐cultivated using a tractor‐mounted offset disc harrow (John Deere, Model 425) and a tractor‐mounted Perfecta field cultivator (Unverferth) to loosen the soil and destroy any winter/spring vegetation before forming raised beds and laying of the plastic mulch. Raised field beds were created using the Kennco superbedder (Kennco Manufacturing Co.). The field beds were 121.9‐m long, 91.4‐cm wide, and 20.3‐cm high. The distance between beds was 3.4 m. Sub‐surface drip irrigation was applied using drip tapes placed centrally underneath the plastic mulch on all raised beds prior to transplanting to maintain adequate soil moisture. The drip tape specifications were 16‐mm hose diameter, 0.20‐mm wall thickness, 30‐cm emitter spacing, with an emitter flow rate of 1.0 L h^−1^ when the pressure regulator was set to 8 psi (Aqua‐Traxx; Toro Agricultural Irrigation). The beds were sprayed with herbicides and the next day they were covered with a black and white totally impermeable film (TIF) plastic mulch (0.03‐mm thick; Polygro LLC) using the Kennco superbedder prior to transplanting.

Seeds for each accession were sown directly into Metro‐Mix 360 soilless media (Sun Gro Horticulture) in 36‐cell vegetable propagation trays (120 cm^3^ cell size; T.O. Plastics Inc.) in the greenhouse at the USVL. Four weeks old seedlings were transplanted by hand to the field plots. In both years, there were three plants per genotype replicated three times. Within genotype spacing, the distance between individual plants was 1.8 m, while the space between genotypes was 2.7 m. Fertilization was conducted via injection into the drip irrigation system with approximately 168 kg ha^−1^ of nitrogen applied over the whole growing season. Weed management was accomplished using a combination of pre‐transplanting herbicide application, plastic mulch, and weekly hand pulling. The experimental design in both 2019 and 2022 field seasons was a randomized complete block design with three replications.

### 2.2 Phenotypic data collection and statistical analyses

Citron watermelon fruits were harvested at physiological maturity based on the presence of a brown and dry tendril at the node bearing the fruit, dull and waxy fruit surface, and light‐colored ground‐spot on the fruit (Maynard, 2001). Majority of the citron watermelon genotypes exhibited a concentrated (uniform) fruit‐set pattern, and fruit harvest for the entire trial was completed over 3 weeks. Three fruits per genotype within each replication were sampled for evaluation of fruit quality traits. Fruit samples were phenotyped based on the fruit quality traits descriptor list for watermelon (UPOV, 2013; Wehner, 2008; Wehner et al., 2001). Fruits were cut open longitudinally (from point of pedicel attachment to blossom end apex) and evaluated for soluble solids content, flesh color, fruit length (distance from pedicel to apex), fruit diameter (distance across widest part of fruit [transverse section] from edge to edge), fruit shape index, and fruit shape. Fruit cores were removed from the center of the cut fruit surface and crushed to extract juice for soluble solids content analysis. A drop of juice from each citron watermelon accession was placed on a hand‐held digital refractometer to record soluble solids content in % Brix (ATAGO). Flesh color was visually scored as red, yellow, orange, green, or white. To assess fruit length and fruit diameter, cut fruits for every genotype were photographed using a Sony Cyber‐shot DSC‐HX400V camera equipped with a 24‐ to 1200‐mm ZEISS lens (SONY). Photographs (RGB images) were then processed using Fiji (ImageJ) software to calculate fruit length and fruit diameter in millimeter (Schindelin et al., 2012). Fruit shape index was computed as the ratio of length to diameter. Fruit shape index distribution was used to delineate fruit shape as round (<1.1), oval (1.1–1.3), or elongate (>1.3) (UPOV, 2013; Wehner, 2008; Wehner et al., 2001).

Statistical analyses on the fruit quality data were performed using PROC MIXED in SAS v9.4 (SAS Institute, 2013). The statistical model for PROC MIXED was given as follows:

$$Y_{ijk}=\mu +G_{i}+S_{j}+GS_{ij}+\mathrm{rep}{\left(S\right)}_{jk}+\varepsilon_{ijk}$$

where *Y_ijk_* is the fruit quality trait value of the *i*th genotype in the *k*th replication of the *j*th field season, *μ* is the grand mean, *G_i_* is the fixed effect of the ith genotype, *S_j_* is the random effect of the *j*th field season, GS*_ij_* is the random interaction term between the *i*th genotype and the *j*th field season, rep(S)*jk* is the random effect of *k*th replication within the jth field season, and *ε_ijk_* is the residual error. The raw traits data were transformed to best linear unbiased estimates (BLUEs) using the restricted maximum likelihood (REML) method in SAS v9.4 (SAS Institute, 2013) based on the above‐mentioned statistical model. The BLUEs were then used as trait input files to execute GWAS. Pearson correlation among traits was performed using PROC CORR in SAS v9.4 (SAS Institute, 2013). Variance components for estimating broad‐sense heritability were calculated using PROC VARCOMP in SAS v9.4 using the REML method (SAS Institute, 2013). Broad‐sense heritability on an entry‐mean basis was calculated using the mathematical formula proposed in Holland et al. (2003) as follows:

$$H^{2}=\frac{\mathrm{Var}\left(G\right)}{\mathrm{Var}\left(G\right)+\frac{\mathrm{Var}\left(\mathrm{GS}\right)}{s}+\frac{\mathrm{Var}\left(\mathrm{Error}\right)}{\mathrm{sr}}}$$

where Var(G) is genotypic variance, Var(GS) is the genotype by field season variance, and Var(Error) is the residual error variance. The denominators *s* and *r* represent the number of field seasons and replications, respectively.

### 2.3 Genotyping, kinship, and population structure

The citron watermelon collection was genotyped using whole‐genome resequencing. Details on genomic DNA isolation, sequencing technologies, and single nucleotide polymorphism (SNP) calling have been presented previously (Katuuramu et al., 2022). Briefly, whole‐genome resequencing data for the 125 citron watermelon (*C*. *amarus*) accessions were obtained using Illumina NovaSeq 6000 sequencing technology (Illumina). Shotgun genomic libraries were sequenced on a single lane using the Illumina NovaSeq 6000 machine to generate 150 bp paired‐end reads. Reads were filtered and trimmed as presented previously in Katuuramu et al. (2022). Variant discovery and quality control were conducted using the GATK v3.6 best practices workflow (DePristo et al., 2011; McKenna et al., 2010; van der Auwera et al., 2013). After filtering for minor allele frequency (≥0.05), a total of 2,126,759 SNPs remained and were used in population structure, relatedness, and marker‐trait association analyses. A kinship matrix to account for relatedness in GWAS was created using the VanRaden method in GAPIT v3.0 within R v4.2.0 (VanRaden, 2008; Wang & Zhang, 2021). Population structure was assessed using neighbor‐joining clustering and principal component analysis (PCA). The phylogenetic tree matrix was created by invoking the neighbor‐joining tree algorithm in TASSEL v5.2 software on the command‐line (Bradbury et al., 2007). The neighbor‐joining dendrogram was displayed using FigTree software (http://tree.bio.ed.ac.uk/software/figtree/). Principal component (PC) analysis to control for population structure was conducted using the *prcomp* function in R v4.2.0 within GAPIT v3.0 (R Core Team, 2022; Wang & Zhang, 2021). The optimum number of PCs to include in the GWAS models for every trait was determined using the scree plot and Bayesian information criterion (Schwarz, 1978).

### 2.4 Genome‐wide association study

Three models were used to perform GWAS and included mixed linear model (MLM), fixed and random model circulating probability unification (FarmCPU), and Bayesian‐information and linkage‐disequilibrium iteratively nested keyway (BLINK) (Huang et al., 2018; Liu et al., 2016; Yu et al., 2006). The MLM is a single‐locus model testing one marker at a time during analysis, while both FarmCPU and BLINK are multiple loci models (Huang et al., 2018; Liu et al., 2016; Yu et al., 2006). MLM model, first published in 2006, incorporates kinship and population structure matrices to control for false negatives and positives. The single‐marker testing makes it computationally intensive and has limited statistical power (Wang & Zhang, 2021; Yu et al., 2006). FarmCPU uses forward‐backward stepwise regression to incorporate multiple markers as covariates to limit confounding markers and kinship (Liu et al., 2016). FarmCPU incorporates fixed‐effects model and a random‐effects model in an iterative fashion resulting in increased statistical power, computational efficiency, and control for both false positives and negatives (Liu et al., 2016; Wang & Zhang, 2021). BLINK runs two fixed effect models: the first one tests for associations between markers and trait values with pseudo quantitative trait nucleotides (QTNs; i.e., previously associated SNP markers with the trait or QTNs) as covariates. In the second step, BLINK optimizes the selection of QTNs to be included as covariates for the first step using Bayesian Information Criterion. BLINK has increased statistical power, is computationally efficient for big genotypic datasets with millions of SNP markers, and better controls both false positives and false negatives compared to the preceding models such as FarmCPU and MLM (Huang et al., 2018; Liu et al., 2016; Wang & Zhang, 2021). Genome‐wide Manhattan and quantile–quantile (QQ) plots to visualize marker‐trait associations and model fitting were created using the *CMplot* package in R v4.2.0 (R Core Team, 2022; Yin, 2022). Model‐trait associations exhibiting irregular deviations in QQ plots are not presented in the results.

Correction for multiple marker‐trait association tests was accomplished using the false discovery rate method at an alpha level of 5% (Benjamin & Hochberg, 1995). Population structure, kinship, and GWAS analyses were implemented in GAPIT v3.0 within R v4.2.0 (Lipka et al., 2012; R Core Team, 2022). The likelihood ratio–based *R* ^2^ statistic was used to calculate the phenotypic variation explained (PVE) by the peak SNP markers for every trait (Sun et al., 2010). Comparison of the allelic effects of the significant SNP markers on the phenotypic means was performed using the Student's two‐tailed *t* test and visualized in R v4.2.0 (R Core Team, 2022). Candidate genes were selected within a ±85 kb interval from the peak SNP for the traits of interest using the released USVL 246‐FR2 *C. amarus* genome (http://cucurbitgenomics.org/). The search window was chosen based on the linkage disequilibrium pattern present in this citron watermelon collection (Katuuramu et al., 2022). Putative candidate genes were those whose annotation, description, and gene ontology functions are relevant to molecular control of the traits evaluated in this research.

## 3 RESULTS

### 3.1 Phenotypic summary statistics, heritability, and trait correlations

**FIGURE 1:**
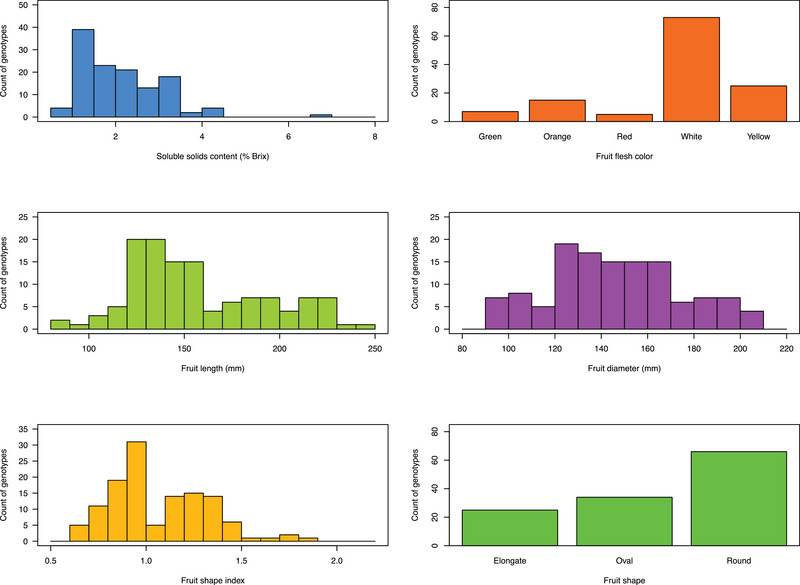
Histogram showing the distribution of soluble solids content, fruit flesh color, fruit length, fruit diameter, fruit shape index, and fruit shape traits across the 125 citron watermelon genotypes averaged over two field growing seasons at the U.S. Vegetable Laboratory research station in Charleston, SC.

Soluble solids content varied from 0.9% to 6.8% Brix with a 7.6‐fold variation and a broad‐sense heritability of 0.97 (Table 1; Figure 1). Five genotypes had a red flesh color, 25 were yellow, 15 had orange flesh, seven were green, and 73 accessions had white flesh color (Figure 1). Fruit flesh color had a 0.96 broad‐sense heritability estimate. Fruit length ranged from 88.5 to 242.9 mm with a 2.7‐fold variation and had a heritability of 0.94 (Table 1; Figure 1). Fruit diameter varied from 90.5 to 209.1 mm with a 2.3‐fold variation and a heritability of 0.89 (Table 1; Figure 1). Fruit shape index ranged from 0.7 to 1.9 with a 2.7‐fold variation and had a heritability estimate of 0.93 (Table 1; Figure 1). Based on the fruit shape index distribution, a total of 25 genotypes had an elongate fruit shape, 34 were oval, and 66 accessions had a round fruit shape (Figure 1). Correlation analysis revealed presence of low to moderate relationships among traits (Table 2). Soluble solids content was positively correlated to fruit length (*r* = 0.50) and fruit diameter (*r* = 0.41; Table 2). Fruit length was positively correlated to fruit diameter (*r* = 0.38) and fruit shape index (*r* = 0.67; Table 2). Fruit diameter was negatively correlated to fruit shape index (*r* = −0.40; Table 2).

**TABLE 1:** Descriptive summary statistics, variance components, and broad‐sense heritability soluble solids content, fruit length, fruit diameter, and fruit shape index traits across the 125 citron watermelon genotypes evaluated over two field seasons at the U.S. Vegetable Laboratory research station in Charleston, SC.

Variables	Soluble solids content (% Brix)	Fruit length (mm)	Fruit diameter (mm)	Fruit shape index
Minimum	0.9	88.5	90.5	0.7
Maximum	6.8	242.9	209.1	1.9
Mean	2.2	157.2	146.2	1.1
Standard deviation	0.9	35.9	28.8	0.3
Fold variation	7.6	2.7	2.3	2.7
Genotypic variance	0.8220	1203.4	738.29	0.0617
Genotype × season interaction variance	0.0063	19.21	93.36	0.0045
Residual error variance	0.1331	337.34	257.63	0.0109
Broad‐sense heritability (*H* ^2^)	0.97	0.94	0.89	0.93

**TABLE 2:** Correlation among soluble solids content, fruit length, fruit diameter, and fruit shape index traits across the 125 citron watermelon genotypes evaluated over two field seasons at the U.S. Vegetable Laboratory research station in Charleston, SC.

Traits	Soluble solids content	Fruit length	Fruit diameter	Fruit shape index
Soluble solids content	–	0.50^*^	0.41^*^	0.15 ns
Fruit length		–	0.38^*^	0.67^*^
Fruit diameter			–	−0.40^*^
Fruit shape index				–

Abbreviation: ns, not significant.

* Significant at the 0.0001 probability level.

### 3.2 Population structure

Neighbor‐joining phylogenetic tree and PCA based on SNP markers were used to determine clustering of the genetic variation present in the *C*. *amarus* collection (Figure S1). There was subtle genetic clustering within the core collection especially since 112 (89.6%) of the genotypes were collected/received from Southern Africa, three were from Asia, five were collected from Europe, and five were received from North America (Table S1; Figure S1). The first and second PCs accounted for 22.1% and 4.7% of the genetic variation present in the *C*. *amarus* core collection, respectively (Figure S1).

### 3.3 Marker‐trait associations and candidate genes

**FIGURE 2:**
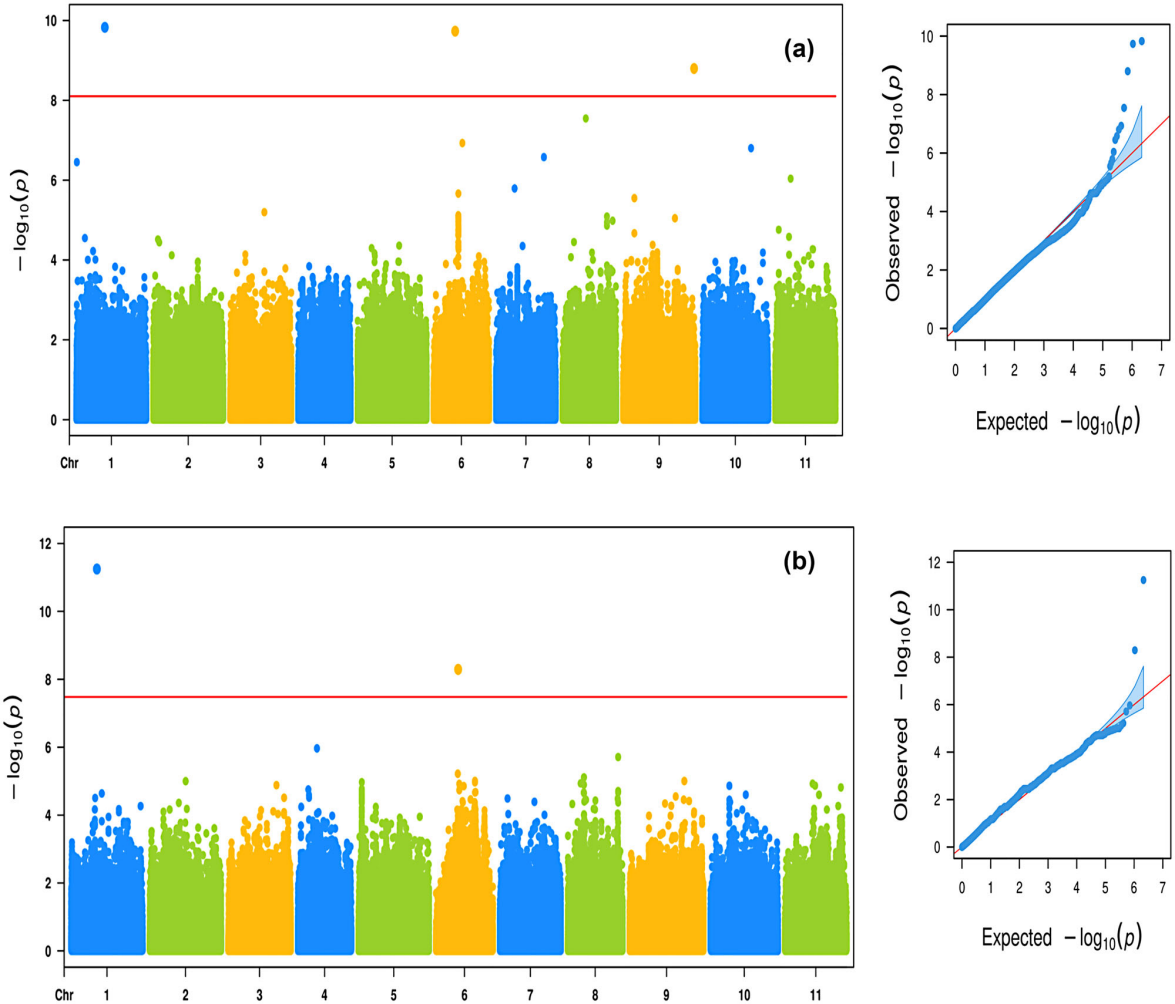
Manhattan and quantile–quantile (QQ) plots for soluble solids content based on (a) fixed and random model circulating probability unification (FarmCPU) and (b) Bayesian‐information and linkage‐disequilibrium iteratively nested keyway genome‐wide association study (BLINK GWAS) models across the 125 citron watermelon genotypes using best linear unbiased estimates (BLUEs) from the two field growing seasons. The red solid horizontal line is a false discovery rate (FDR) cutoff of *α* = 0.05.

**FIGURE 3:**
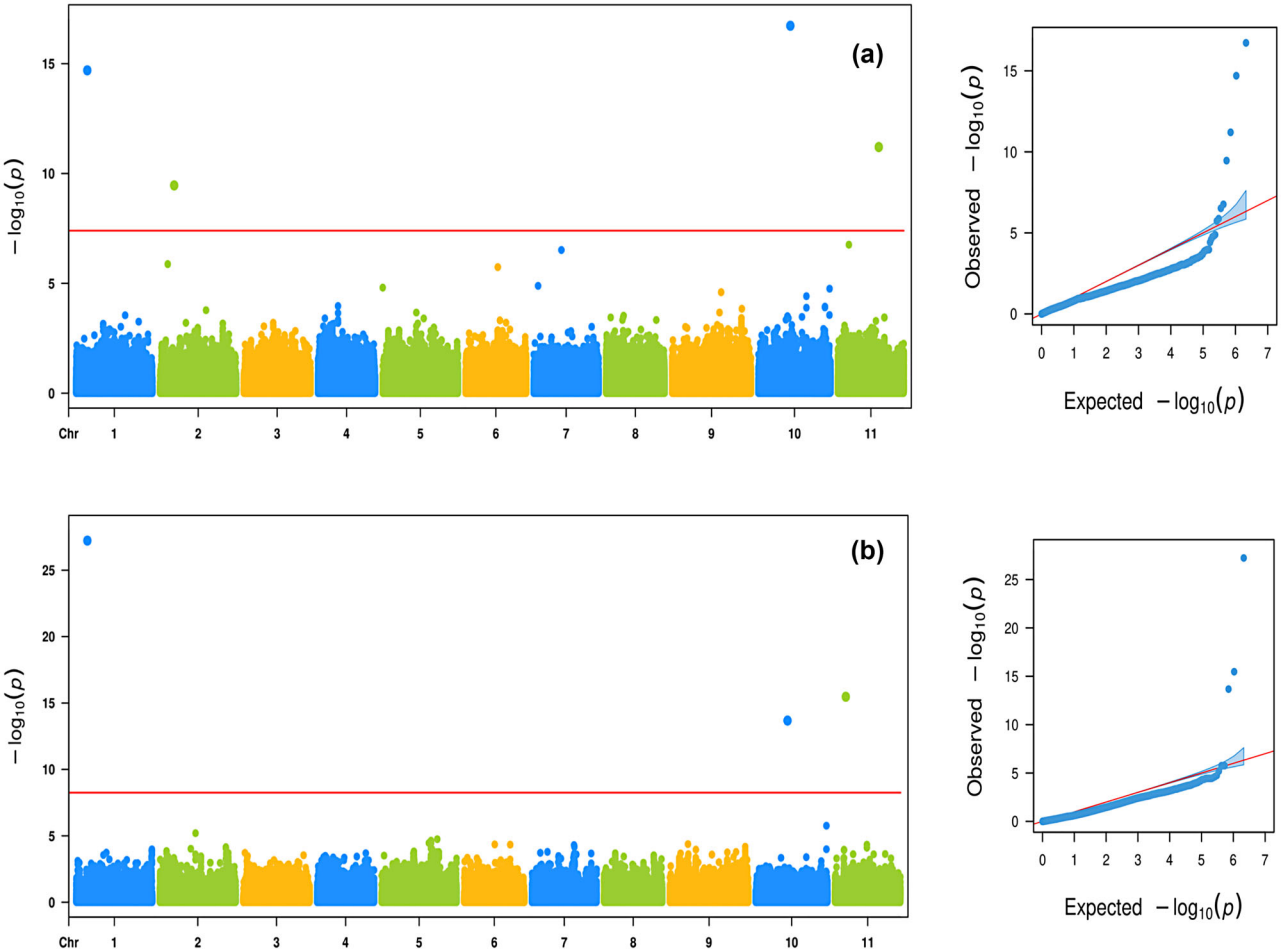
Manhattan and quantile–quantile (QQ) plots for fruit flesh color based on (a) fixed and random model circulating probability unification (FarmCPU) and (b) Bayesian‐information and linkage‐disequilibrium iteratively nested keyway genome‐wide association study (BLINK GWAS) models across the 125 citron watermelon genotypes using best linear unbiased estimates (BLUEs) from the two field growing seasons. The red solid horizontal line is a false discovery rate (FDR) cutoff of *α* = 0.05.

Four SNP markers were significantly associated with soluble solids content (*p* ≤ 1.6 × 10^−9^) and were located on chromosomes Ca01, Ca06, and Ca09 (Table 3; Figure 2). The SNP marker on chromosome Ca06 (S6_11610218) was uncovered by both BLINK and FarmCPU models (Table 3; Figure 2). These four peak SNPs explained 2.7%–22.4% of the phenotypic variation in soluble solids content (Table 3). Five peak SNPs were associated with fruit flesh color (*p* ≤ 3.5 × 10^−10^) and located on chromosomes Ca01, Ca02, Ca10, and Ca11 (Table 3; Figure 3). Two of the peak SNPs on chromosomes Ca01 (S1_5486012) and Ca10 (S10_15722135) were uncovered by both BLINK and FarmCPU models (Table 3; Figure 3). The five significant SNPs explained 2.9%–13.7% of the phenotypic variation in fruit flesh color (Table 3).

**TABLE 3:** Details of the peak single nucleotide polymorphisms (SNPs) associated with soluble solids content, flesh color, fruit length, fruit diameter, and fruit shape index traits across the 125 citron watermelon genotypes evaluated over two field seasons at the U.S. Vegetable Laboratory research station in Charleston, SC.

Traits	Chr	SNP	SNP position (bp)	SNP *p* value	MAF	Major allele	Minor allele	PVE (%)	GWAS model
Soluble solids content	Ca01	S1_13515500	13,515,500	5.6E‐12	0.22	A	G	2.7	BLINK
	Ca01	S1_15333555	15,333,555	1.5E‐10	0.17	T	G	6.0	FarmCPU
	Ca06	S6_11610218	11,610,218	1.9E‐10	0.30	C	T	22.4	BLINK, FarmCPU
	Ca09	S9_38139503	38,139,503	1.6E‐09	0.23	C	T	4.3	FarmCPU
Flesh color	Ca01	S1_5486012	5,486,012	2E‐15	0.35	C	T	13.7	BLINK, FarmCPU
	Ca02	S2_6968474	6,968,474	3.5E‐10	0.44	A	G	2.9	FarmCPU
	Ca10	S10_15722135	15,722,135	1.9E‐17	0.24	G	T	11.5	BLINK, FarmCPU
	Ca11	S11_5350066	5,350,066	3.4E‐16	0.14	T	G	12.7	BLINK
	Ca11	S11_20091563	20,091,563	6.2E‐12	0.09	C	T	11.4	FarmCPU
Fruit length	Ca03	S3_30466135	30,466,135	1.9E‐09	0.44	G	A	20.1	MLM
	Ca03	S3_30466436	30,466,436	4.6E‐20	0.43	C	T	19.1	BLINK, FarmCPU, MLM
	Ca03	S3_30466640	30,466,640	3.1E‐09	0.44	G	C	19.5	MLM
	Ca10	S10_17320207	17,320,207	2.2E‐08	0.36	C	G	21.5	BLINK, FarmCPU
Fruit diameter	Ca01	S1_27855991	27,855,991	1.7E‐10	0.22	G	T	2.6	FarmCPU
	Ca02	S2_6968517	6,968,517	4.7E‐09	0.12	C	T	2.5	FarmCPU
	Ca02	S2_8370640	8,370,640	3.8E‐09	0.48	C	T	2.9	BLINK
	Ca03	S3_30466135	30,466,135	3.2E‐09	0.44	G	A	19.0	MLM
	Ca03	S3_30466436	30,466,436	1.6E‐36	0.43	C	T	11.7	BLINK, FarmCPU, MLM
	Ca03	S3_30466640	30,466,640	8.3E‐09	0.44	G	C	17.9	MLM
	Ca07	S7_2706920	2,706,920	2.8E‐10	0.13	G	A	4.9	FarmCPU
	Ca08	S8_405281	405,281	1.8E‐09	0.35	G	A	5.7	FarmCPU
	Ca08	S8_12874511	12,874,511	6.2E‐26	0.45	C	T	10.4	FarmCPU
	Ca08	S8_13477026	13,477,026	6.8E‐10	0.34	T	C	19.4	BLINK
Fruit shape index	Ca03	S3_30461306	30,461,306	1.2E‐09	0.44	G	A	22.3	MLM
	Ca03	S3_30462710	30,462,710	3.6E‐09	0.21	C	A	20.9	MLM
	Ca03	S3_30462870	30,462,870	5.4E‐09	0.35	A	T	19.9	MLM
	Ca03	S3_30464456	30,464,456	5.3E‐09	0.42	A	C	19.4	MLM
	Ca03	S3_30466135	30,466,135	3.7E‐15	0.44	G	A	23.4	MLM
	Ca03	S3_30466436	30,466,436	7.5E‐51	0.43	C	T	19.2	BLINK, FarmCPU, MLM
	Ca03	S3_30466640	30,466,640	8.5E‐15	0.44	G	C	21.1	MLM
	Ca04	S4_3713000	3,713,000	1.7E‐09	0.27	C	T	2.5	FarmCPU
	Ca04	S4_5755109	5,755,109	1.6E‐10	0.29	G	A	2.4	FarmCPU
	Ca05	S5_4283299	4,283,299	1.7E‐09	0.29	C	T	2.5	FarmCPU
	Ca07	S7_5980465	5,980,465	1.7E‐11	0.13	G	A	9.4	BLINK, FarmCPU
	Ca07	S7_9869290	9,869,290	1.2E‐09	0.14	A	G	2.1	FarmCPU
	Ca08	S8_7694448	7,694,448	7.8E‐13	0.10	A	T	5.3	FarmCPU
	Ca10	S10_5269197	5,269,197	1.7E‐11	0.17	C	T	3.6	FarmCPU

Abbreviations: BLINK, Bayesian‐information and linkage‐disequilibrium iteratively nested keyway; Chr, citron watermelon chromosome; FarmCPU, fixed and random model circulating probability unification; GWAS, genome‐wide association study; MAF, minor allele frequency; MLM, mixed linear model; PVE, phenotypic variation explained.

**FIGURE 4:**
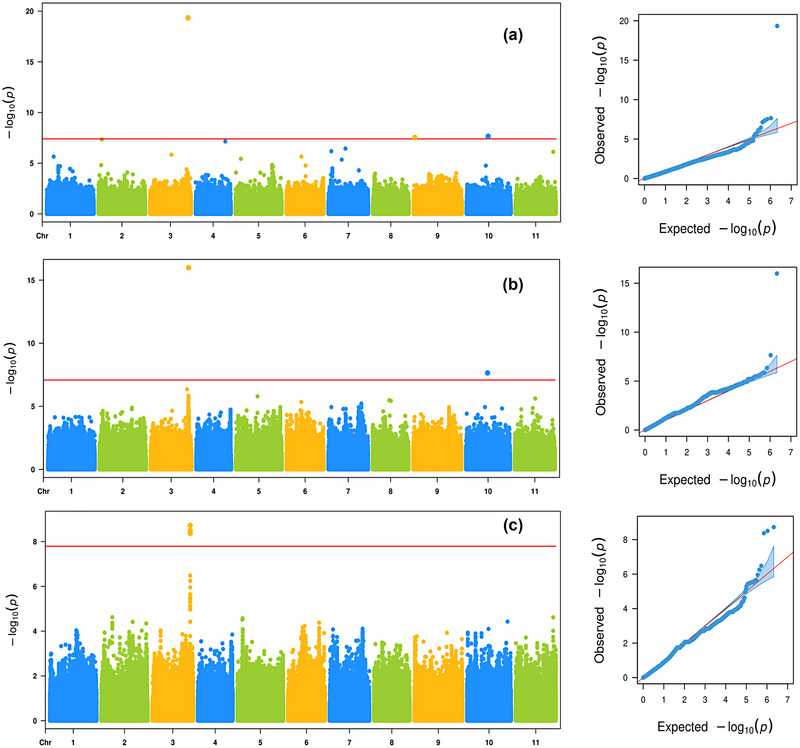
Manhattan and quantile–quantile (QQ) plots for fruit length based on (a) fixed and random model circulating probability unification (FarmCPU), (b) Bayesian‐information and linkage‐disequilibrium iteratively nested keyway (BLINK), and (c) mixed linear model genome‐wide association study (MLM GWAS) models across the 125 citron watermelon genotypes using best linear unbiased estimates (BLUEs) from the two field growing seasons. The red solid horizontal line is a false discovery rate (FDR) cutoff of *α* = 0.05.

**FIGURE 5:**
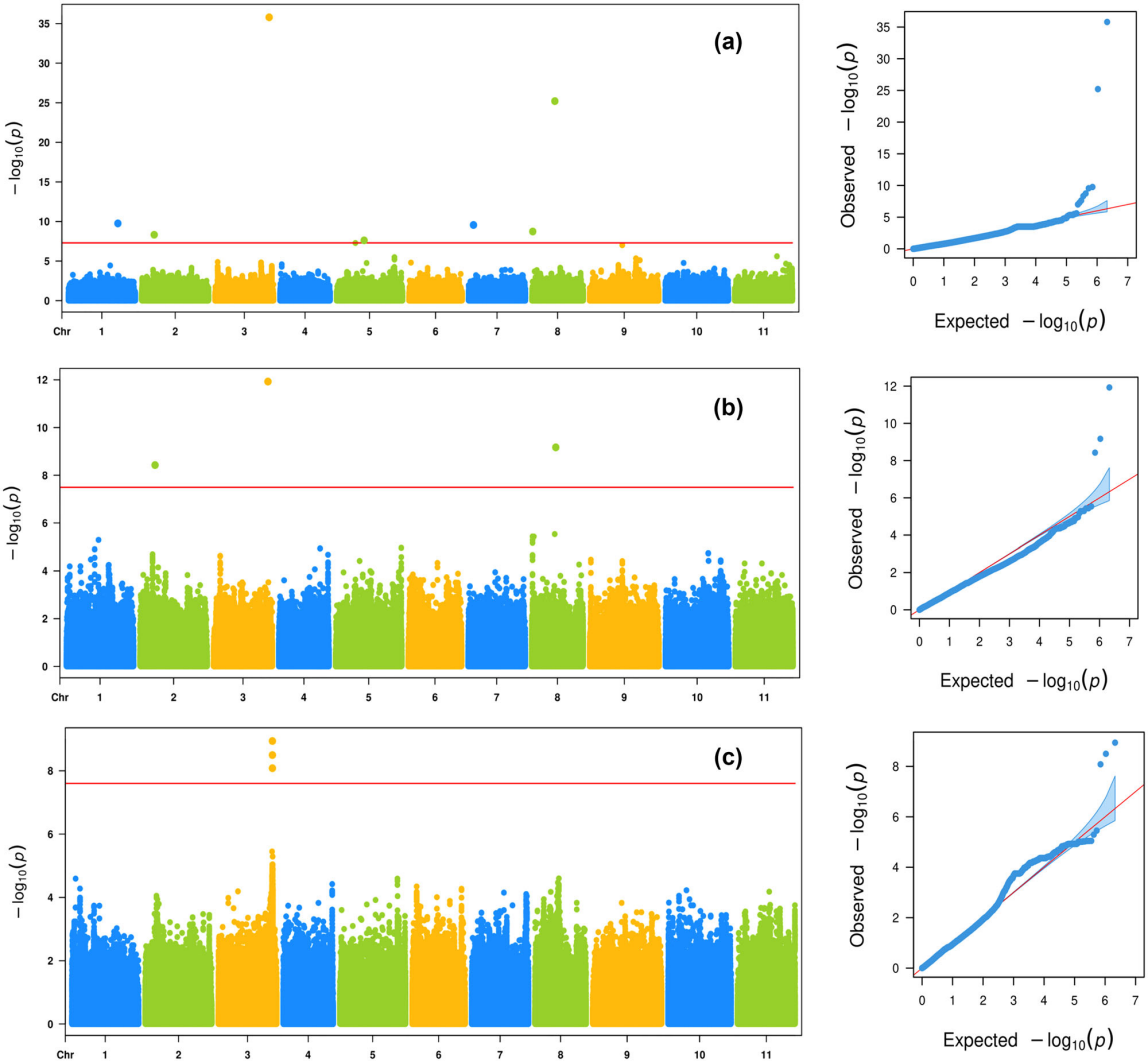
Manhattan and quantile–quantile (QQ) plots for fruit diameter based on (a) fixed and random model circulating probability unification (FarmCPU), (b) Bayesian‐information and linkage‐disequilibrium iteratively nested keyway (BLINK), and (c) mixed linear model genome‐wide association study (MLM GWAS) models across the 125 citron watermelon genotypes using best linear unbiased estimates (BLUEs) from the two field growing seasons. The red solid horizontal line is a false discovery rate (FDR) cutoff of *α* = 0.05.

**FIGURE 6:**
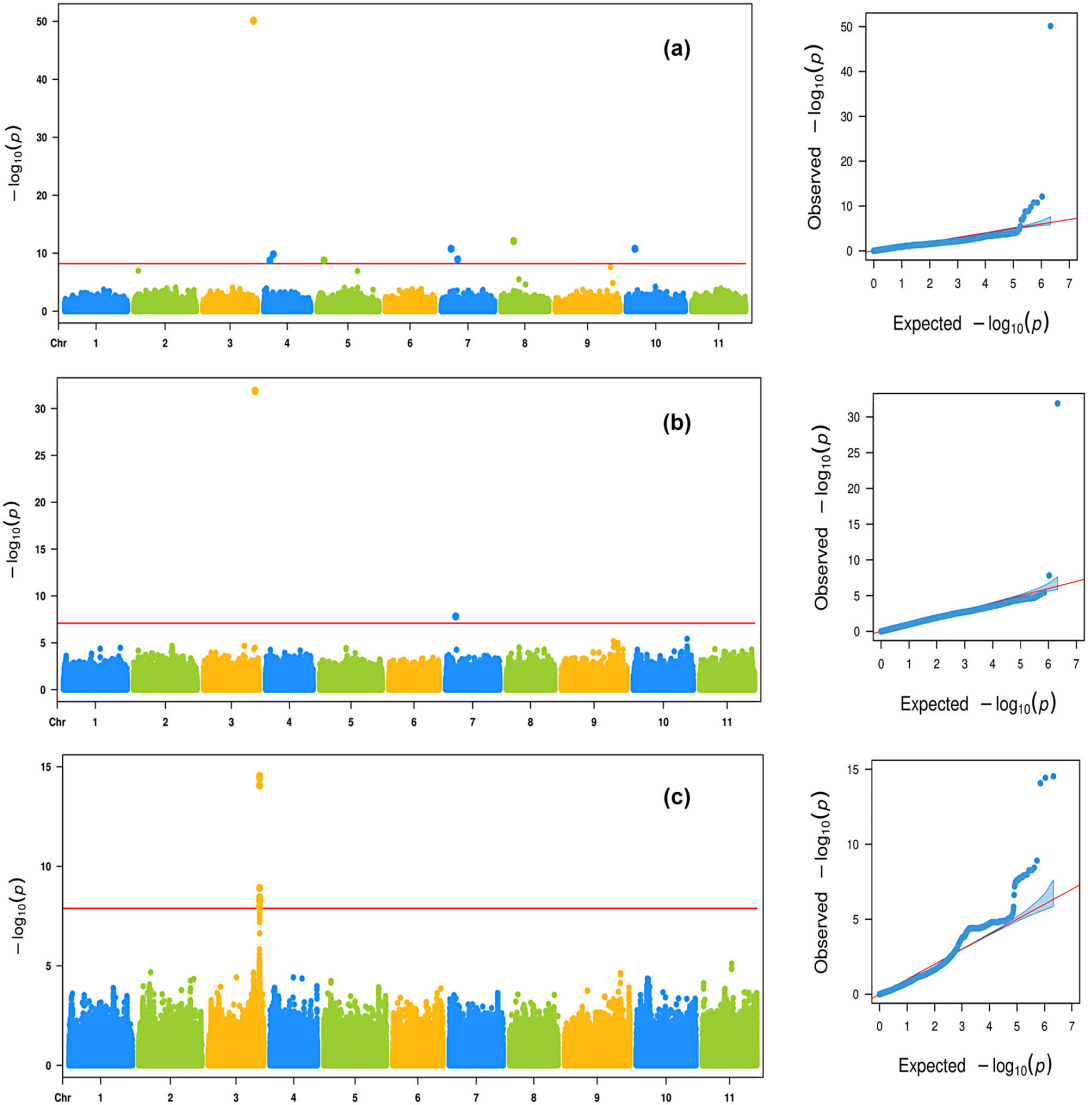
Manhattan and quantile–quantile (QQ) plots for fruit shape index based on (a) fixed and random model circulating probability unification (FarmCPU), (b) Bayesian‐information and linkage‐disequilibrium iteratively nested keyway (BLINK), and (c) mixed linear model genome‐wide association study (MLM GWAS) models across the 125 citron watermelon genotypes using best linear unbiased estimates (BLUEs) from the two field growing seasons. The red solid horizontal line is a false discovery rate (FDR) cutoff of *α* = 0.05.

There were four significant SNPs associated with fruit length (*p* ≤ 2.2 × 10^−8^) and were located on chromosomes Ca03 and Ca10 (Table 3; Figure 4). The most significant SNP marker (S3_30466436) on chromosome Ca03 was uncovered by BLINK, FarmCPU, and MLM models (Table 3; Figure 4). Another peak SNP on chromosome Ca10 (S10_17320207) was uncovered by both BLINK and FarmCPU models (Table 3; Figure 4). The PVE by the peak SNPs for fruit length ranged from 19.1% to 21.5% (Table 3). Ten significant SNP markers were associated with fruit diameter (*p* ≤ 8.3 × 10^−9^) and were located on chromosomes Ca01, Ca02, Ca03, Ca07, and Ca08 (Table 3; Figure 5). The peak SNP marker (S3_30466436) on chromosome Ca03 was uncovered by BLINK, FarmCPU, and MLM models (Table 3; Figure 5). The 10 significant SNPs explained 2.5%–19.4% of the phenotypic variation in fruit diameter (Table 3). There were 14 significant SNPs associated with fruit shape index (*p* ≤ 5.4 × 10^−9^) and were located on chromosomes Ca03, Ca04, Ca05, Ca07, Ca08, and Ca10 (Table 3; Figure 6). The significant SNP marker (S3_30466436) on chromosome Ca03 was uncovered by BLINK, FarmCPU, and MLM models (Table 3; Figure 6). Another peak SNP marker (S7_5980465) was uncovered by both BLINK and FarmCPU models (Table 3; Figure 6). These 14 significant SNPs explained 2.1%–23.4% of the phenotypic variation in fruit shape index (Table 3).

Candidate genes relevant to sugar transport and accumulation as well as fruit development and fruit shape were identified. Candidate gene *CaU06G09710* was located at 83.9 kb downstream of the peak SNP S6_11610218 associated with soluble solids content on chromosome Ca06 and encodes for a sugar transporter. A significant SNP S3_30466436 located on chromosome Ca03 was associated with fruit length, fruit diameter, and fruit shape index (Table 3). This SNP was consistently detected by the three GWAS models of BLINK, FarmCPU, and MLM. The SNP was located inside candidate gene *CaU03G18580* that codes for an IQ domain protein. Candidate gene *CaU01G17460* was located at 82.5 kb downstream of the significant SNP S1_27855991 associated with fruit diameter on chromosome Ca01 and codes for WD40 repeat protein. Another peak SNP S7_2706920 associated with fruit diameter is co‐located with candidate gene *CaU07G02850*. The *CaU07G02850* candidate gene was found at 22.6 kb downstream of the significant SNP and codes for a squamosa promoter binding protein.

### 3.4 Allelic effects of the significant SNPs on the fruit quality traits

**FIGURE 7:**
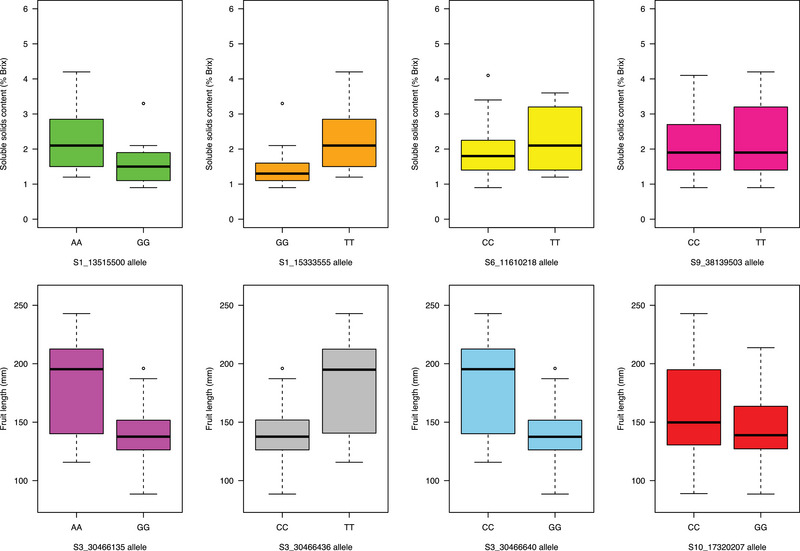
Boxplot showing allelic effects of the peak single nucleotide polymorphism (SNP) markers associated with soluble solids content and fruit length traits in the 125 citron watermelon genotypes based on Bayesian‐information and linkage‐disequilibrium iteratively nested keyway (BLINK), fixed and random model circulating probability unification (FarmCPU), and mixed linear model genome‐wide association study (MLM GWAS) models.

**FIGURE 8:**
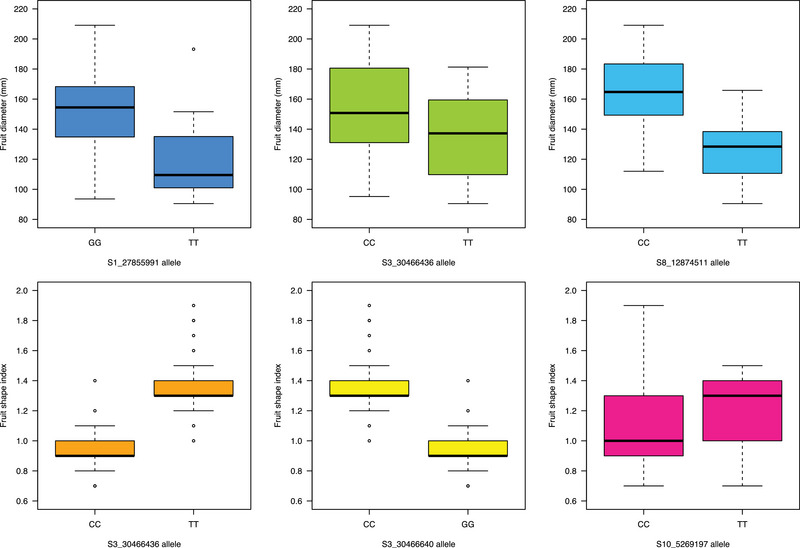
Boxplot showing allelic effects of the peak single nucleotide polymorphism (SNP) markers associated with fruit diameter and fruit shape index traits in the 125 citron watermelon genotypes based on Bayesian‐information and linkage‐disequilibrium iteratively nested keyway (BLINK), fixed and random model circulating probability unification (FarmCPU), and mixed linear model genome‐wide association study (MLM GWAS) models.

Allelic effects of the peak SNP markers on the fruit quality traits were assessed (Figures 7 and 8). For the “AA” allele of SNP marker S1_13515500 on chromosome Ca01 resulted in higher soluble solids content (mean = 2.2% Brix) compared with a mean of 1.6% Brix among genotypes with the homozygous “GG” alternative allele (*p* = 9.7 × 10^−4^; Figure 7). Genotypes with the GG allele for SNP marker S1_15333555 on chromosome Ca01 had a lower mean soluble solids content of 1.5% Brix compared to accessions carrying the “TT” allele with a mean of 2.2% Brix (*p* = 5.3 × 10^−5^; Figure 7). For the “CC” and TT alleles of SNP markers S6_11610218 and S9_38139503 on chromosomes Ca06 and Ca09, there were no significant differences in soluble solids content (Figure 7). For fruit flesh color, SNP marker S1_5486012 on chromosome Ca01, the major allelic class CC was predominantly associated with white flesh color (56.5%), while genotypes with the minor allele TT had mostly orange or yellow flesh colors (Table S2). The major allele AA of SNP S2_6968474 on chromosome Ca02 was associated mostly with white fleshed accessions (43.7%), while the minor allele GG was associated with yellow (14.6%) and white (16.5%) fruit color phenotypes. The major allele GG of SNP marker S10_15722135 on chromosome Ca10 was largely associated with white flesh color, while minor allele TT was associated with orange (10.5%) and yellow (8.9%) flesh colors (Table S2). The TT major allele of SNP S11_5350066 on chromosome Ca11 was largely associated with white flesh color (52.4%), while genotypes carrying the GG minor allele were mostly yellow and white fleshed. For SNP marker S11_20091563 on chromosome Ca11, genotypes with the CC major allele were predominantly associated with white flesh color (58.5%), while accessions carrying the minor allele TT were mostly yellow fleshed (Table S2).

For fruit length, genotypes with the AA allele for SNP marker S3_30466135 on chromosome Ca03 had longer fruits (mean = 180.7 mm) compared with the GG allele (mean = 138.9 mm; *p* = 3.1 × 10^−9^; Figure 7). The CC allele of SNP marker S3_30466436 on chromosome Ca03 resulted in shorter fruits (mean = 139.4 mm) compared to the TT allele with a mean of 180.5 mm at a *p* value of 2.9 × 10^−9^ (Figure 7). For SNP marker S3_30466640, genotypes with the CC allele had longer fruits (mean = 180.7 mm) compared to accessions with the GG allele (mean = 138.7 mm; *p* = 2.9 × 10^−9^; Figure 7). A haplotype combination involving allelic states AA, TT, and CC of S3_30466135, S3_30466436, and S3_30466640 SNP markers, respectively, on chromosome Ca03 corresponded to longer fruit length, and there were 40 accessions carrying the AATTCC haplotype in the citron watermelon germplasm collection. For alleles CC and GG of SNP marker S10_17320207 on chromosome Ca10, there was no significant difference in fruit length (Figure 7).

For fruit diameter, the GG allele of SNP marker S1_27855991 on chromosome Ca01 resulted in larger diameter values (mean = 153.6 mm) compared to the TT allele (mean = 120.1 mm; *p* = 3.8 × 10^−6^; Figure 8). Accessions with the CC allele of SNP marker S3_30466436 on chromosome Ca03 had larger fruit diameter readings (mean = 154.3 mm) compared to accessions carrying the TT allele (mean = 135.4 mm; *p* = 3.1 × 10^−4^; Figure 8). Genotypes with the CC allele for SNP S8_12874511 had larger fruit diameter readings (mean = 165.5 mm) compared to those with the TT allele (mean = 126.5 mm; *p* = 2.8 × 10^−17^; Figure 8). For fruit shape index, the CC allele for SNP marker S3_30466436 on chromosome Ca03 resulted in smaller value (mean = 0.8) compared to the TT allele (mean = 1.4; *p* = 9.7 × 10^−25^; Figure 8). Genotypes with the CC allele for SNP marker S3_30466640 on chromosome Ca03 had a higher fruit shape index value (mean = 1.4) compared to accessions with the GG allele (mean = 0.9; *p* = 5.3 × 10^−24^; Figure 8). For the CC and TT alleles of SNP marker S10_5269197 on chromosome Ca10, there were no significant differences in fruit shape index values (Figure 8).

## 4 DISCUSSION

Improving fruit quality traits is one of the major goals for commercial and public watermelon breeding programs. Consumer choice and repeat purchasing of watermelon produce is greatly influenced by fruit sweetness, flesh color, and fruit shape (Kaiser, 2012; Maynard, 2001). The screening of the citron watermelon collection revealed variations within the fruit quality traits. High broad‐sense heritability was detected for all traits suggesting presence of genetic contributions to the observed variation. High heritability estimates for soluble solids content and flesh color have been reported previously (Ren et al., 2014; Wehner et al., 2017). Significant correlations were identified that ranged from low to moderate indicating that the traits could be combined in multiple ways during mating designs for fruit quality improvement. The positive correlation among soluble solids content, fruit length, and fruit diameter suggests that it is feasible to increase flesh sweetness while simultaneously selecting for larger fruit size in watermelon breeding and cultivar development. Previous research efforts have also presented positive correlations among soluble solids content, fruit length, and fruit diameter traits in a field‐based watermelon trial (Correa et al., 2020). Fruit diameter was negatively correlated to fruit shape index implying that increasing diameter resulted in fruits with round fruit shape or conversely shorter fruit diameter resulted in elongate fruit shape.

Many SNPs (37) significantly associated with soluble solids content, flesh color, fruit length, fruit diameter, and fruit shape index were detected using BLINK, FarmCPU, and MLM models, and they appear to confer small genetic effects with PVE ≤ 23.4%. The small effects of the peak SNPs highlight the complexity of the genetics underlying the evaluated fruit quality traits. Utilization of these three models in the analysis helped account for differences in the underlying phenotypic variation and genetic architecture of the fruit quality traits. The MLM method was limited in the number of traits where significant marker‐trait associations were detected. No peak SNPs were found for soluble solids content and fruit flesh color when using MLM. This can be attributed to the low statistical power and poor control of false negatives common to MLM method compared to multiple‐locus models such as BLINK and FarmCPU (Huang et al., 2018; Liu et al., 2016; Wang & Zhang, 2021; Yu et al., 2006). There was concordance across models where five significant SNP markers were detected by BLINK and FarmCPU over all fruit quality traits. One SNP marker significant for fruit length, diameter, and fruit shape index was detected by MLM, BLINK, and FarmCPU models. This consistency across models suggests that these genomic regions are crucial in the genetic control of the fruit quality traits. Identification of molecular markers that influence fruit quality traits and understanding the genetic mechanism will be crucial in marker‐assisted trait improvement. Genomic loci underlying soluble solids content and flesh color on chromosomes 2, 4, 5, and 10 in *C*. *lanatus* have been reported (Fall et al., 2019; Guo et al., 2019; Wu et al., 2019). The present research uncovered loci for soluble solids and flesh color on chromosomes Ca01, Ca02, Ca06, Ca09, Ca10, and Ca11. The new peak SNPs uncovered in this research will be useful in marker‐assisted fruit quality trait improvement in watermelon.

Genomic loci associated with fruit shape traits (length, diameter, and fruit shape index) were uncovered on chromosomes Ca01, Ca02, Ca03, Ca04, Ca05, Ca07, Ca08, and Ca10. Previous studies reported genomic regions for fruit shape on chromosomes 1, 2, 3, and 6 (Dou et al., 2018; Guo et al., 2019; Wu et al., 2019). All the peak SNPs for fruit length on chromosome Ca03 were located at approximately 30.5 Mb and were also associated with fruit diameter and fruit shape index including the most significant SNP suggesting presence of shared genetic control for these three traits. Guo et al. (2019) reported that five SNPs on chromosome 3 (at approximately 31.1 Mb) were associated with fruit shape in *C. lanatus*. This region on chromosome Ca03 warrants further investigation since it had peak SNPs consistently detected by BLINK, FarmCPU, and MLM models and co‐located with a fruit shape candidate gene.

Four candidate genes relevant to fruit sweetness (measured by soluble solids content) and fruit shape traits (length, diameter, and fruit shape index) co‐located with the peak SNPs generated from GWAS. The functional annotation of the candidate genes was as follows: *CaU06G09710* (sugar transporter), *CaU03G18580* (IQ domain protein), *CaU01G17460* (WD40 repeat protein), and *CaU07G02850* (squamosa promoter binding protein). Translocation and accumulation of sugars from the source (e.g., photosynthetically active tissues) to sink organs such as fruits relies heavily on sugar transporter proteins (Kuhn & Grof, 2010; Williams et al., 2000). Moreover, overexpression of a tonoplast sugar transporter in watermelon resulted in elevated sugar levels in the fruit (Ren et al., 2018). The peak SNP markers and sugar transporter gene will be useful in manipulation of the fruit sink strength ultimately improving fruit sweetness demanded by watermelon consumers.

Three candidate genes (*CaU03G18580* [IQ domain protein], *CaU01G17460* [WD40 repeat protein], and *CaU07G02850* [squamosa promoter binding protein]) co‐located with the peak SNPs associated with fruit length, diameter, and fruit shape index. In watermelon fruit, development processes involve elongation and enlargement. The IQ domain proteins play crucial roles in plant organ development and shape as well as tolerance to drought stress (Duan et al., 2017; Lazzaro et al., 2018). Previous GWAS and integrated omics reports showed IQ domain proteins to be involved in fruit shape and elongation in watermelon and tomatoes (Dou et al., 2022, 2018; Guo et al., 2019; Rodriguez et al., 2011). The WD40 repeat proteins play important roles in many plant development processes including cell division, floral development, and meristem organization (Stirnimann et al., 2010). Squamosa promoter binding proteins play critical roles in plant architecture, flower and fruit development, and fruit ripening among other plant developmental phase transitions across many plant species (Chen et al., 2010). These candidate genes warrant further extensive analysis to determine their molecular and functional mechanisms in the control of fruit quality traits.

## 5 CONCLUSION

In this study, extensive phenotyping of fruit quality traits in a citron watermelon collection and trait mapping using GWAS were performed. Significant variation for fruit quality traits was detected in the citron watermelon germplasm. Results from GWAS revealed genomic loci underlying fruit quality traits. Candidate genes relevant to fruit quality traits and co‐located with the significant SNPs are provided. Phenotypic variation will be useful in parental selections for crossing blocks to improve watermelon fruit quality traits. The significant SNP markers and candidate genes will be crucial in development of molecular tools to improve watermelon fruit quality traits via marker‐assisted breeding.

## AUTHOR CONTRIBUTIONS

**Dennis N. Katuuramu**: Conceptualization; methodology; investigation; data curation; formal analysis; visualization; writing—original draft; writing—review and editing. **Amnon Levi**: Conceptualization; resources; writing—review and editing. **William P. Wechter**: Conceptualization; resources; writing—review and editing.

## CONFLICT OF INTEREST STATEMENT

The authors declare no conflicts of interest.

## Supporting information

Supplementary Figure S1. Population structure assessment based on (A) neighbor joining phylogenetic tree and (B) principal component analysis in the citron watermelon collection. tpg220391‐sup‐0002‐TableS1.xlsx

Supplementary Table S1. Geographical origin of the 125 citron watermelon accessions evaluated for soluble solids content, flesh color, and fruit shape traits.
SUPPLEMENTARY TABLE S2 Allelic effects of the peak single nucleotide polymorphism (SNP) markers associated with fruit flesh color in the citron watermelon germplasm collection based on BLINK and FarmCPU GWAS models.

## Data Availability

The original contributions presented in this research are available within the article and the supplementary files. The genomic datasets used in this research can be accessed at different websites: http://cucurbitgenomics.org/; http://cucurbitgenomics.org/ftp/genome/watermelon/USVL246/; http://cucurbitgenomics.org/ftp/reseq/watermelon/Amarus_reseq/. Any reasonable inquiries/requests can be made to the corresponding author.
