# Supplementary material for: Genome‐wide association study of soluble solids content, flesh color, and fruit shape in citron watermelon
Source: Plant Genome. 2023 Sep 18;16(4):e20391. doi: 10.1002/tpg2.20391 (PMC12807390; doi:10.1002/tpg2.20391)
Supplement: Supplementary file 1 [file TPG2-16-e20391-s001.pdf]

The Plant Genome Journal

Supplementary Materials For:

Genome-wide association study of soluble solids content, flesh color, and fruit shape in citron watermelon

Authors: Dennis N. Katuuramu, Amnon Levi, and William P. Wechter

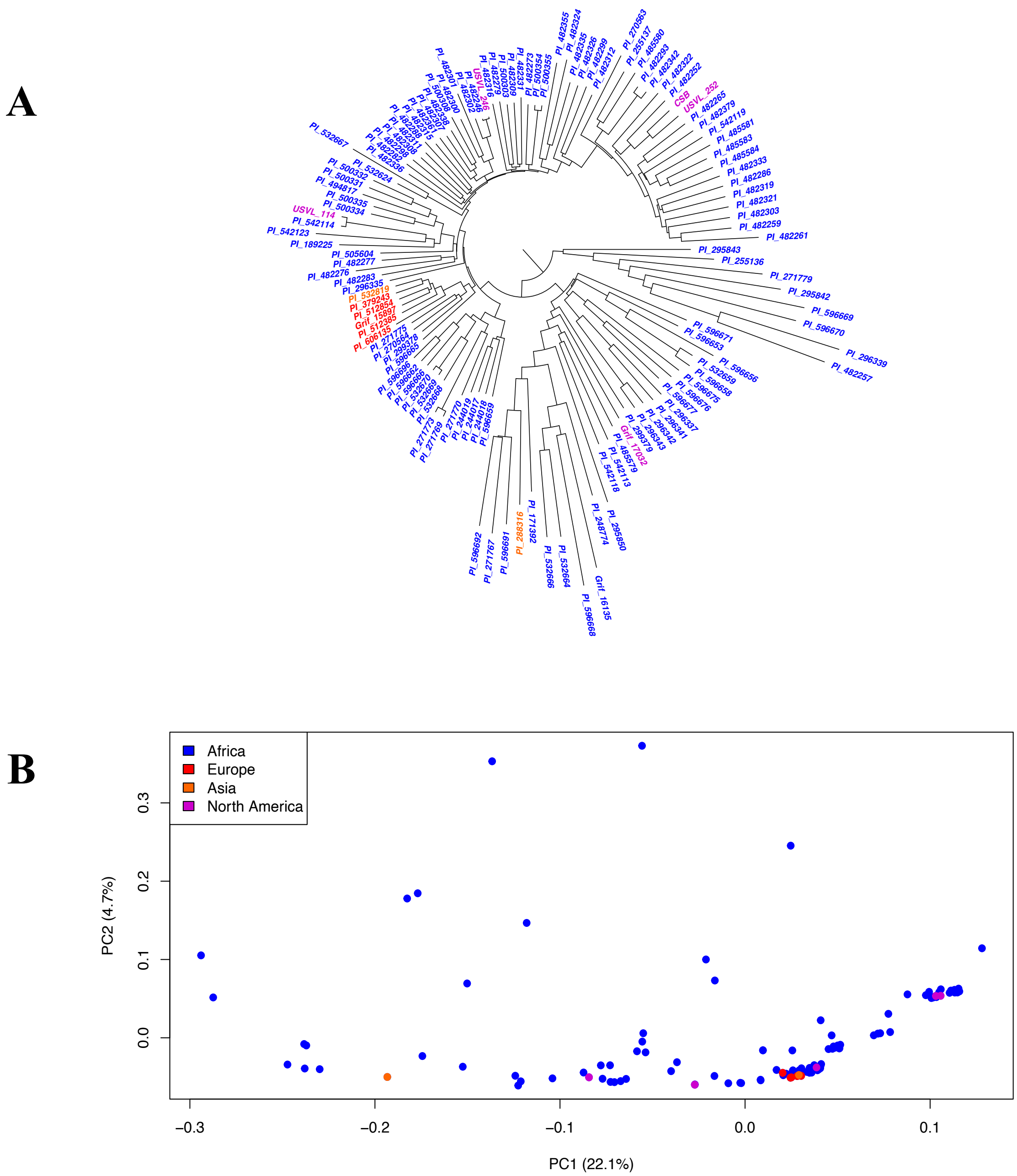

SUPPLEMENTARY FIGURE S1 Population structure assessment based on (**A**) neighbor-joining phylogenetic tree and (**B**) principal component analysis in the citron watermelon collection.

SUPPLEMENTARY TABLE S2 Allelic effects of the peak SNP markers associated with fruit flesh color in the citron watermelon germplasm collection based on BLINK and FarmCPU GWAS models.

Fruit flesh color	SNP marker ID[x]									
	S1_5486012		S2_6968474		S10_15722135		S11_5350066		S11_20091563	
	Major allele	Minor allele	Major allele	Minor allele	Major allele	Minor allele	Major allele	Minor allele	Major allele	Minor allele
	C	T	A	G	G	T	T	G	C	T
Green	4 (3.2)	3 (1.7)	3 (2.9)	2 (1.9)	5 (4.0)	2 (1.6)	5 (4.0)	2 (1.6)	6 (4.9)	1 (0.8)
Orange	0 (0)	15 (8.3)	4 (3.9)	7 (6.8)	2 (1.6)	13 (10.5)	14 (11.3)	1 (0.8)	9 (7.3)	6 (4.9)
Red	1 (0.8)	3 (1.7)	1 (1.0)	3 (2.9)	3 (2.4)	1 (0.8)	2 (1.6)	2 (1.6)	3 (2.4)	0 (0)
White	70 (56.5)	3 (1.7)	45 (43.7)	17 (16.5)	69 (55.6)	4 (3.2)	65 (52.4)	8 (6.5)	72 (58.5)	1 (0.8)
Yellow	5 (4.0)	20 (11.1)	6 (5.8)	15 (14.6)	14 (11.3)	11 (8.9)	20 (16.1)	5 (4.0)	12 (9.8)	13 (10.6)

[x] Numbers before the brackets in each column are genotype counts having a particular flesh color within the major or minor allelic classes for the corresponding significant SNP marker. Numbers inside the brackets are genotype percentages for a given peak SNP marker.
